# Randomised phase II evaluation of irinotecan plus high-dose 5-fluorouracil and leucovorin (ILF) *vs* 5-fluorouracil, leucovorin, and etoposide (ELF) in untreated metastatic gastric cancer

**DOI:** 10.1038/sj.bjc.6602649

**Published:** 2005-06-07

**Authors:** M Moehler, A Eimermacher, J Siebler, T Höhler, A Wein, M Menges, D Flieger, T Junginger, T Geer, E Gracien, P R Galle, M Heike

**Affiliations:** 1 Klinikum der Johannes-Gutenberg-Universität, Mainz, Dortmund, Germany; 2 Universtitätsklinik Erlangen, Erlangen, Dortmund, Germany; 3 Universitätskliniken des Saarlandes, Homburg/Saar, Dortmund, Germany; 4 Klinikum Aschaffenburg, Aschaffenburg, Dortmund, Germany; 5 Diakonie Krankenhaus, Schwäbish Hall, Dortmund, Germany; 6 Aventis Pharma Deutschland GmbH, Bad Soden/Ts; 7 Med. Department Mitte, Klinikum Dortmund gGmbH, Beurhausstr. 10, Dortmund 44137, Germany

**Keywords:** ELF, 5-fluorouracil, gastric, ILF, irinotecan, metastatic

## Abstract

An open-label randomised comparison of efficacy and tolerability of irinotecan plus high-dose 5-fluorouracil (5-FU) and leucovorin (LV) (ILF) with etoposide plus 5-FU/LV (ELF) in patients with untreated metastatic or locally advanced gastric cancer. One cycle of ILF comprised six once-weekly infusions of irinotecan 80 mg m^−2^, LV 500 mg m^−2^, 24-h 5-FU 2000 mg m^−2^, and ELF comprised three once-daily doses of etoposide 120 mg m^−2^, LV 300 mg m^−2^, 5-FU 500 mg m^−2^. In all, 56 patients received ILF and 58 ELF. Median age was 62 years, Karnofsky performance 90%, and disease status was comparable for both arms. The objective clinical response rates after 14 weeks treatment (primary end point) were 30% for ILF and 17% for ELF (risk ratio (RR) 0.57, 95% confidence interval (CI) 0.29–1.13, *P*=0.0766). Overall response rates over the entire treatment period for ILF and ELF were 43 and 24%, respectively (RR 0.56, 95% CI 0.33–0.97; *P*=0.0467). For ILF and ELF, respectively, median progression-free survival was 4.5 *vs* 2.3 months, time to treatment failure was 3.6 *vs* 2.2 months (*P*=0.4542), and overall survival was 10.8 *vs* 8.3 months (*P*=0.2818). Both regimens were well tolerated, the main grade 3/4 toxicities being diarrhoea (18%, ILF) and neutropenia (57%, ELF). The data from this randomised phase II study indicate that ILF provides a better response rate than ELF, and that ILF should be investigated further for the treatment of metastatic gastric cancer.

Gastric cancer is the fourth most common cancer in Europe and the third leading cause of cancer mortality (Bray *et al*, 2002). Although gastric cancer has declined over the past 50 years, the incidence of tumours at the gastro-oesophageal junction has increased (Blot *et al*, 1991). The use of chemotherapy for the management of patients with advanced gastric cancer, who have limited treatment options (Hohenberger and Gretschel, 2003), has only become widely acceptable over the last 20 years (Glimelius *et al*, 1997; Murad *et al*, 1993; Pyrhonen *et al*, 1995).

5-Fluorouracil (5-FU), usually in combination with leucovorin (LV, also referred to as folinic acid), forms the basis of most chemotherapy regimens used for the treatment of gastric cancer. A randomised phase III trial compared combinations of 5-FU with other active drugs in advanced gastric cancer: etoposide, LV and 5-FU (ELF) *vs* infusional 5-FU plus cisplatin (FUP) *vs* 5-FU, doxorubicin and methotrexate (FAMTX) (Vanhoefer *et al*, 2000). The overall response rates (ORRs) ranged from 9% (ELF) to 20% (FUP) and median survival times were between 6.7 months (FAMTX) and 7.2 months (both ELF and FUP). The observed differences were not statistically significant and there is still no definitive regimen for the treatment of gastric cancer. The combination of epirubicin, cisplatin and continuous infusion 5-FU (ECF) has been proposed as a standard first-line therapy for gastric cancer as a consequence of its significantly improved response rate (46%) and survival (8.7 months) when compared with FAMTX (Waters *et al*, 1999). More recently, ELF has been shown to provide better disease control (complete response (CR)+partial response (PR)+stable disease (SD)) for patients with proximal rather than distal tumours (85 *vs* 48%, *P*=0.04) (Schulze-Bergkamen *et al*, 2002). Tolerability, toxicity and ease of administration have become major determinants for selecting an appropriate therapy and ELF has emerged as a convenient, well-tolerated regimen that can be administered on an outpatient basis (Vanhoefer *et al*, 2000; Schulze-Bergkamen *et al*, 2002).

Irinotecan (CPT-11, Camptosar; Pfizer Oncology, New York, USA) inhibits topoisomerase I thereby disrupting DNA replication and cell division within tumour cells. Response rates between 20 and 23% have been reported for irinotecan monotherapy in untreated gastric cancer (Futatsuki *et al*, 1994; Kohne *et al*, 2003). In patients who had failed previous therapy, irinotecan (180 mg m^−2^) combined with 5-FU (400 mg m^−2^, bolus) and LV (125 mg m^−2^) followed by 5-FU infusion (1200 mg m^−2^ over 48 h) yielded a response rate of 29%, while a further 34% of patients achieved SD (Assersohn *et al*, 2004). Irinotecan with bolus-LV/5-FU in the first-line treatment of gastric cancer provided a response rate of 22% (Blanke *et al*, 2001). However, this regimen (irinotecan 125 mg m^−2^, LV 20 mg m^−2^ plus 5-FU 500 mg m^−2^, all given weekly for 4 weeks followed by a 2-week rest) was associated with a high incidence of severe diarrhoea (28%) and neutropenia (36%) infection leading to substantial dose modifications (Blanke *et al*, 2001). By comparison, the combination of irinotecan with continuous rather than bolus infusions of LV/5-FU exhibited a lower incidence of grade 3 and 4 toxicities in colorectal cancer patients (Douillard *et al*, 2000; Saltz *et al*, 2000; Bouzid *et al*, 2003). Therefore, we have investigated a weekly dose of irinotecan (80 mg m^−2^) in combination with LV (500 mg m^−2^) and continuous 5-FU (2000 mg m^−2^ over 24 h) according to the AIO (Arbeitsgemeinschaft Internistische Onkologie) regimen (i.e. ILF) in gastric cancer patients. In a previous phase I study of ILF in the first- and second-line treatment of gastric cancer, we observed a response rate of 20% with a further 36% of patients reporting SD (Moehler *et al*, 2003). Importantly, toxicity was sufficiently manageable to allow outpatient-based treatment. Therefore, we initiated the present randomised, controlled, phase II study to compare the efficacy and safety of ILF with ELF in the first-line treatment of metastatic gastric cancer.

## PATIENTS AND METHODS

### Patients

Eligible patients had untreated histologically proven gastric adenocarcinoma, or adenocarcinoma of the oesophagogastric junction with measurable metastatic disease and/or locally recurrent nodal involvement, were aged between 18 and 75 years with a Karnofsky performance score (KPS) ⩾60 and a life expectancy >12 weeks. Patients were required to have adequate haematological (neutrophils ⩾2.0 × 10^9^ l^−1^, platelets ⩾150 × 10^9^ l^−1^; haemoglobin ⩾10g dl^−1^), hepatic (total bilirubin ⩽1.25 × upper normal limit (UNL); aspartate (AST) and alanine (ALT) aminotransferases ⩽3 × UNL) and renal function (creatinine <1.25 × UNL). Patients with previous cancer therapies were excluded from the study.

All patients provided signed and dated consent before entering the trial. The study was conducted in accordance with the principles of the Declaration of Helsinki and Good Clinical Practice guidelines and the protocol was initially approved by the Ethics committee of Aerztekammer Rheinland-Pfalz and later by all Ethics committees responsible for participating centres.

### Study design and randomisation

This was an open-label, multicentre, phase II randomised trial with two treatment arms. Patients were randomly assigned and stratified according to centre, peritoneal involvement (yes/no) and prior gastrectomy (yes/no). The randomisation process was centralised and performed by the Coordination Centre for Clinical Trials (KKS), Mainz, Germany.

### Administration of study drugs and dose adjustment

Patients assigned to ILF (Arm A) received irinotecan 80 mg m^−2^ intravenously (i.v.) over 60–90 min followed by LV 500 mg m^−2^ i.v. over 60 min and then 5-FU 2000 mg m^−2^ i.v. over 24 h, on day 1. Each cycle comprised six once-weekly treatments followed by a 13-day rest period. Systemic prophylactic atropine (0.25 mg) injections for irinotecan-related acute cholinergic symptoms were allowed for the first cycle but not recommended. Prophylactic treatment for delayed diarrhoea was not permitted. However, patients were carefully informed of the potential risk of delayed diarrhoea and neutropenia and the need for early intervention with loperamide, metoclopramide, antibiotics, or hospitalisation and parenteral rehydration in case of refractory diarrhoea (>48 h). Antiemetic treatment was performed using metoclopramide or HT-3 antagonists in a sequential manner. The prophylactic use of colony-stimulating factors was not permitted.

Patients assigned to ELF (Arm B) received etoposide 120 mg m^−2^ i.v. over 60 min, LV 300 mg m^−2^ i.v. over 5–10 min and then 5-FU 500 mg m^−2^ bolus i.v. over 2–4 min, on day 1. Each cycle comprised three applications on consecutive days (1–3) followed by an 18-day rest.

All study treatments were administered until disease progression, unacceptable toxicity or withdrawal of consent. In the event of toxicity (defined by the National Cancer Institute of Canada expanded common toxicity criteria; NCIC-CTC), treatment delays or dose reductions could be applied as follows. If at any time during a cycle there were moderate reductions in haematological function (neutrophil count 0.5–1.5 × 10^9^ l^−1^, platelet count 25–75 × 10^9^ l^−1^) or moderate diarrhoea or stomatitis (>grade 1), the next administration could be delayed for up to 2 weeks. If at any time haematological abnormalities were noted (neutrophils <0.5 × 10^9^ l^−1^, neutrophils <1 × 10^9^ l^−1^ with infection or fever, platelets <25 × 10^9^ l^−1^) or if there were ⩾grade 3 or 4 diarrhoea or stomatitis, treatment had to be delayed until recovery to moderate levels (as described above) after which the following dose reductions were applied. For Arm A, 5-FU was reduced to 1600 mg m^−2^ and irinotecan to 65 mg m^−2^. For Arm B, in the case of haematological toxicity, etoposide had to be reduced to 100 mg m^−2^ and 5-FU to 400 mg m^−2^, and in the case of diarrhoea or stomatitis, 5-FU was reduced to 400 mg m^−2^. If a condition persisted despite dose reduction, or if a patient experienced myocardial infarction, treatment was terminated. In the case of hand–foot syndrome, the dose of 5-FU was to be reduced by 20%. Delayed diarrhoea was treated immediately with loperamide and rehydration and, if associated with severe neutropenia, a broad-spectrum antibiotic. Hospitalisation with i.v. rehydration was required for grade 4 or persistent (>48 h) diarrhoea, concomitant vomiting, fever, or KPS <60%.

### Study evaluations

At baseline up to five measurable lesions per organ and 10 lesions in total were to be identified as target lesions, measured using computed tomography (CT), and recorded according to the RECIST system (Response Evaluation Criteria In Solid Tumours; Therasse *et al*, 2000). The sum of the longest diameters for all target lesions was used as a reference for determining objective tumour response. Tumour responses were evaluated at week 7, week 14 and then every two cycles for patients receiving ILF or every four cycles for patients receiving ELF. Responses were determined according to RECIST as follows: complete response was defined as the disappearance of all target and nontarget lesions with no new lesions and confirmed by two observations at least 4 weeks apart; PR was defined as a reduction of 30% or more in the sums of the longest diameters of all measurable lesions relative to baseline with no new lesions; no change (NC) was defined as neither sufficient shrinkage to qualify for PR nor sufficient increase to qualify for progressive disease (PD) with no new lesions; and PD was defined as ⩾20% increase in the sum of the longest diameters, the occurrence of nontarget lesions (e.g. pleural effusion or ascites) or the appearance of brain metastases independently of performance at sites outside the brain.

Safety and tolerability were assessed by regular clinical examinations and assessments of adverse events (weekly, at the end of treatment and at every 3 months of follow-up), disease symptoms, KPS, haematological and biochemical parameters.

### Statistical analysis

The primary end point was objective clinical response (CR+PR) based on an interim analysis following 14 weeks of treatment. The one-sided exact Fisher's test was used to compare the treatment arms at the significance level *α*=5%. The analyses were performed on an intention-to-treat basis including all patients who were treated in the study.

The secondary end points were ORR (for the entire treatment period), time to progression, tumour growth control, time to treatment failure (including progression, death or withdrawal) and survival.

Time to event data were described by Kaplan–Meier estimates and treatment groups were compared by log-rank test. Time to event data were further evaluated by appropriate proportional Cox's models and results were summarised by hazard ratio point and 95% confidence interval (CI) estimates, and *P*-values of Wald *χ*^2^ test. Binary data were described by risk ratio (RR) point and 95% CI estimates and treatment groups were compared by exact Fisher's test. Binary data were further evaluated by appropriate logistic regression models and were summarised by odds ratio point and 95% CI estimates and *P*-values of Wald *χ*^2^ test. If not specified otherwise, *P*-values are presented from two-sided tests and two-sided 95% CI are presented. All analyses were performed using SAS version 6.12.

## RESULTS

### Patient characteristics

In all, 120 patients from 17 centres in Germany were randomised into the study between November 2000 and April 2003. Two patients from Arm A and four from Arm B withdrew without receiving study treatment; therefore, the intention-to-treat population contained 114 patients (56 received ILF and 58 received ELF). The baseline characteristics were well balanced between the two treatment groups (Table 1). The median age of patients was 62 years and the median KPS was 90%. In around one-third (31%) of patients, the primary tumour site was the oesophagogastric junction, 62% of patients had liver metastases and in 77% two or more organs were involved.

### Response rates

The objective clinical response rates following 14 weeks of treatment (primary end point) were 30% for ILF compared with 17% for ELF (RR 0.57, 95% CI 0.29–1.13, *P*=0.0766). The ORRs for the entire treatment period and including all assessments prior to discontinuation were 43% (24 of the 56) for ILF and 24% (14 of the 58) for ELF (Table 2). The increased response rate provided by ILF compared with ELF was statistically significant (RR=0.56; 95% CI=0.33–0.97; *P*=0.0467.) The tumour control rates (CR+PR+NC) were 63% (35 of the 56) and 41% (24 of the 58), respectively.

Logistic regression analysis indicated that a baseline KPS ⩽80% reduced the likelihood of a response by 59% compared with patients whose KPS was greater than 80% (*P*=0.038) (Table 3). After adjustment for KPS, peritoneal involvement and surgery for primary tumour, the regression model also demonstrated that ILF was 138% more likely to provide a response when compared with the ELF regimen (Table 3, *P*=0.042).

### Progression-free survival, treatment failure and overall survival

At the last data cutoff, the median follow-up was 9.4 months in Arm A and 5.8 months in Arm B. At this time, 96 of the 114 patients had died. Disease progression was the major cause of death and accounted for 79% of patients in both treatment groups. One patient from the ILF arm did not comply with the provided recommendations for the treatment of prolonged grade 3 diarrhoea and consequently died (i.e. toxic death). One patient in the ELF arm died from a cardiovascular event.

**Figure 1:**
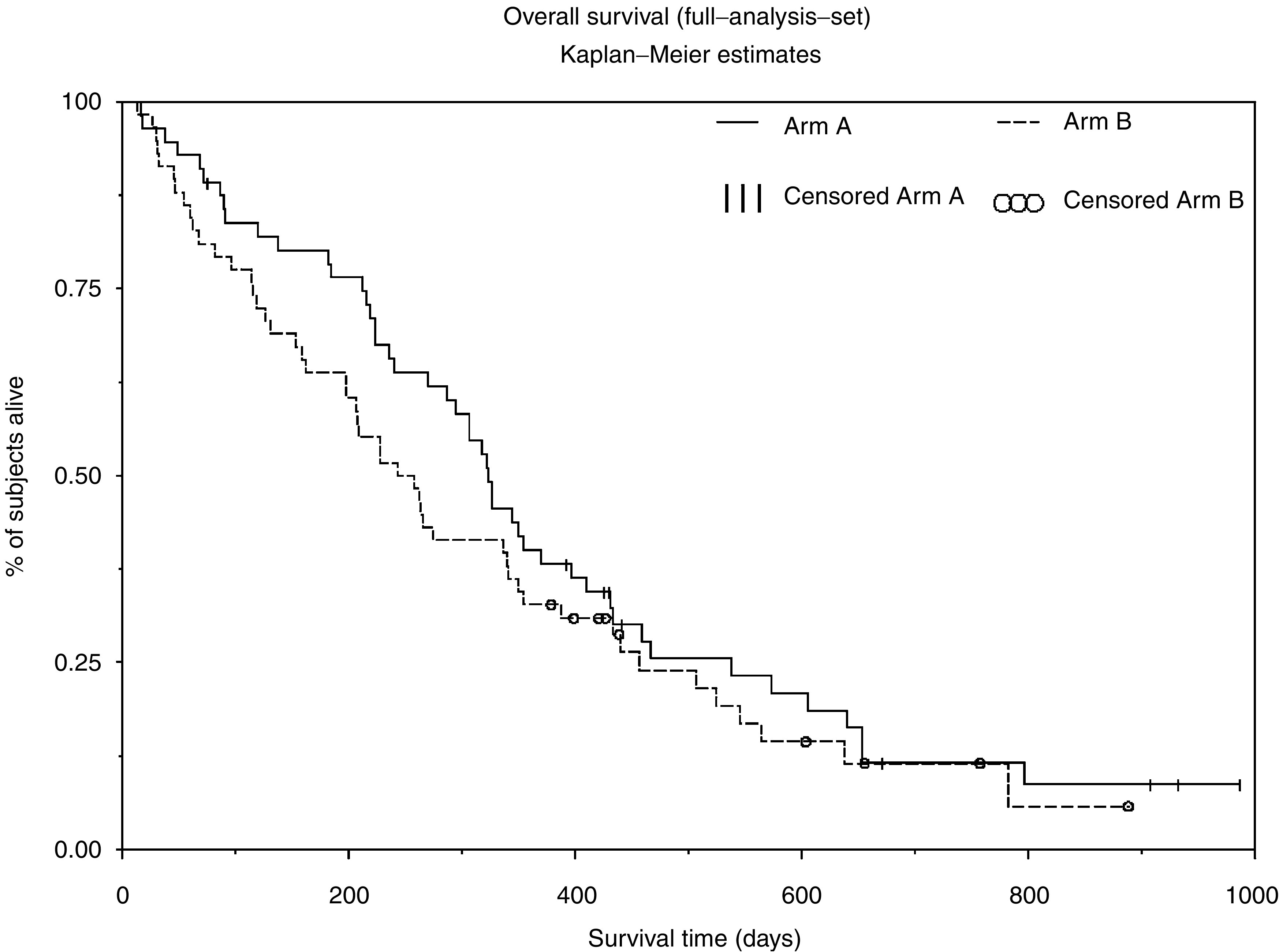
Overall survival. Arm A=irinotecan+5-fluorouracil/leucovorin (ILF), *N*=56. Arm B=etoposide+5-fluorouracil/leucovorin (ELF), *N*=58.

Compared with ELF, the ILF regimen extended median progression-free survival, median time to treatment failure and median overall survival (Table 4). However, when the treatment groups were compared by log-rank test, there was no significant difference between the two treatments for any of these parameters (e.g. the Kaplan–Meier survival plot as shown in Figure 1).

Investigational analyses found that the risk of progression was increased in patients with a primary tumour in the oesophagogastric junction and in those with metastatic involvement in two or more organs (Table 3). As would be expected, the risk of death was increased in patients with a low KPS, in those with two or more involved organs and in those with peritoneal involvement who received ELF (Table 3).

### Safety

The median number of cycles administered in the study was two for ILF (Arm A) and three for ELF (Arm B) (Table 5). Although the median treatment duration period was over twice as long with ILF than with ELF, there were more dose administration delays (70%) and dose reductions (75%) with ILF than with ELF (52 and 45%, respectively). The main reason for discontinuing study treatment was disease progression; 54% of patients receiving ILF and 72% receiving ELF. Although only one patient in each treatment group withdrew because of treatment-related toxicity, five patients receiving ILF and three patients receiving ELF either withdrew consent or refused further treatment.

The incidence of grade 3/4 haematological toxicities was low in both treatment groups with the exception of neutropenia, which was reported by 57% of patients receiving ELF (Table 6). There were more grade 3/4 gastrointestinal toxic events with ILF, notably diarrhoea, which was reported by 18% of patients compared with no reports with ELF. Grade 3/4 alopecia was reported by a significant proportion of patients receiving ELF (28%), but was only seen in 5% of those receiving ILF.

## DISCUSSION

Although chemotherapy regimens offer at best a slight, albeit statistically significant, improvement in survival for patients with gastric cancer, they are associated with a degree of toxicity that limits their value as a palliative treatment (Vanhoefer *et al*, 2000; Schoffski, 2002; Diaz-Rubio, 2004).

The primary end point of clinical response at 14 weeks was selected so that a statistical comparison at a fixed time point could be made. However, as it is the convention in such studies, patients were treated until progression, and could respond to treatment at a later point. Therefore, the overall response and survival rates obtained from the entire dosing period provide a more clinically significant assessment of the efficacy of these regimens for discussion in relation to other trials in gastric cancer. The irinotecan-based combination provided again a greater ORR than that seen with the commonly used ELF regimen (43 *vs* 24%, respectively, *P*=0.0467).

Overall response rates for ELF reported in previous studies range from 9 to 23% (Vanhoefer *et al*, 2000; Schulze-Bergkamen *et al*, 2002) and this compares well with the 24% response rate reported in this study. Accordingly, an ORR of nearly 50% for ILF, as seen in this study, is a substantial improvement and is in the range of previous reports of the use of this drug combination in this setting (Blanke *et al*, 2001; Moehler *et al*, 2003).

The overall survival data in the present study also compare well with those from previous studies. The median overall survival with ELF has been reported at 7.2 and 8.0 months (Vanhoefer *et al*, 2000; Schulze-Bergkamen *et al*, 2002), which is similar to both the 8.3 months reported here and the data reported for irinotecan-based regimens in the second-line setting between 7.0 and 7.6 months (Moehler *et al*, 2003; Assersohn *et al*, 2004). By comparison, there was a nonsignificant trend for increased median survival with ILF in this study (10.8 months) and this compares well with data reported for more recent exploratory combinations such as capecitabine and docetaxel (10.5 months), and epirubicin, docetaxel and cisplatin (11.0 months) (Lee *et al*, 2004; Park *et al*, 2004). The same can be said of the progression-free survival period in the ILF group of 4.5 months, which compared well with the 4.1 and 5.2 months reported recently for docetaxel-based regimens (Lee *et al*, 2004; Park *et al*, 2004). In other randomised phase II studies, continuous 5-FU/LV infusion plus irinotecan has also provided promising efficacy (ORRs of 40–42%, median progression-free survival periods of 6.5–6.9 months and median overall survival periods of 10.7–11.3 months; Bouche *et al*, 2004; Pozzo *et al*, 2004). Consequently, large phase III studies are being considered to investigate irinotecan in combination with continuous 5-FU/LV infusion regimens.

When patient histories, disease status and other factors were examined for their effects on clinical outcome, those patients who were in better general health (good performance status, low tumour burden) were more likely to achieve a response and less likely to have a progression event or die, regardless of the treatment arm to which they were randomised. Patients with peritoneal involvement at presentation have a generally poorer prognosis and as a group face a desperate need for improved treatment options (Rau *et al*, 1996). The data from this study demonstrated that these patients are less likely to suffer a fatal event if treated with ILF rather than ELF. This is potentially an important observation for the management of these difficult to treat patients (Blot *et al*, 1991; Bray *et al*, 2002).

The extension of meaningful survival remains a major objective for oncologists who must therefore consider the impact of treatment-related toxicity. Overall, the occurrence of the toxicities in this study was consistent with the safety profiles of irinotecan, etoposide and 5-FU/LV. The ILF combination was well tolerated with a low and acceptable incidence of haematological toxicity. Gastrointestinal toxicity is a recognised side effect of ILF therapy (Douillard *et al*, 2000; Saltz *et al*, 2000), which can require hospitalisation and urgent medical intervention (Rothenberg *et al*, 2001). The incidence of grade 3 or 4 diarrhoea in the current study was comparable to the previous data in gastric cancer (Blanke *et al*, 2001; Moehler *et al*, 2003; Pozzo *et al*, 2004). With close monitoring of the patient, suitable medication and rehydration, most cases of diarrhoea can be managed effectively and do not present a significant obstacle to the clinical use of ILF.

The toxicity observed in our study was lower than that reported by Douillard *et al* in the pivotal European first-line trial where patients with colorectal cancer received weekly irinotecan (80 mg m^−2^) plus an AIO-based regimen of 24-h high-dose 5-FU (2300 mg m^−2^) preceded by 2-h LV 500 mg m^−2^, and grade 3/4 diarrhoea was reported by 44% of patients (Douillard *et al*, 2000). The lower toxicity in our study might be due to the lower daily doses of 5-FU (2000 mg m^−2^ administered over 24 h). Work is ongoing to identify those patients who carry a specific genetic polymorphism in one of the main enzymes (UGT1A1) involved in the detoxification of irinotecan and are therefore more susceptible to the side effects of irinotecan (Mathijssen *et al*, 2001). Such work will improve the targeting of this useful therapy and may allow appropriate prescriptive dosing schedules on an individual basis.

The present study concurs with similar phase II studies in that the combination of irinotecan with continuous LV/5-FU (ILF) represents a potentially valuable new treatment option for metastatic gastric cancer and requires further evaluation (Bouche *et al*, 2004; Pozzo *et al*, 2004).

## Figures and Tables

**Table 1:** Patient and disease characteristics at baseline (intention-to-treat population)

	**Irinotecan+5- FU/LV**	**Etoposide+5- FU/LV**
No. of exposed patients	56	58

*Gender (n (%))*		
Male	40 (71)	49 (85)
Female	16 (29)	9 (16)
		
*Age (years)*		
Median (range)	61 (41–73)	63 (34–76)
		
*KPS*		
Median value	90	85
		
*Distribution (n (%))*		
60+70	3 (5)	7 (12)
80	21 (38)	22 (38)
90	28 (50)	21 (36)
100	4 (7)	8 (14)
		
*Primary tumour location (n (%))*		
Stomach	37 (66)	42 (72)
Oesophagogastric junction	19 (34)	16 (28)
		
*Median time (months (range)) since*		
Histological confirmation	1.5 (0–181)	1.4 (0–65)
Diagnosis of metastasis	0.7 (0–12)	0.5 (0–13)
		
*Prior surgery*		
Number with surgery (%)	29 (52)	31 (53)
Median months since surgery (range)	7.7 (0–181)	11.1 (0–65)
		
*Location of metastases at entry (n (%))*		
Skin	0	1 (2)
Liver	34 (61)	37 (64)
Lung	12 (21)	5 (9)
Lymph nodes	40 (71)	47 (81)
Bone marrow	0	0
Bone	4 (7)	1 (2)
Peritoneum	10 (18)	11 (19)
Other	20 (36)	23 (40)
		
*Number of involved organs (n (%))*		
1	13 (23)	13 (22)
2	25 (45)	25 (43)
3	15 (27)	18 (31)
4	3 (5)	2 (3)

KPS=Karnofsky performance score.

**Table 2:** Response to therapy in the entire treatment period

	**Number (%) of patients**	
**Response category**	**Irinotecan+5-FU/LV (*N*=56)**	**Etoposide+5-FU/LV (*N*=58)**
CR	2 (4)	0
PR	22 (39)	14 (24)
NC	11 (20)	10 (17)
PD	13 (23)	27 (47)
Missing	8 (14)	7 (12)
		
Overall response (CR+PR)	24 (43)	14 (24)
		
	RR (95% CI), *P*-value	
	0.56 (0.33–0.97), *P*=0.0467	
Disease control (CR+PR+NC)	35 (63)	24 (41)

5-FU=5-fluorouracil; LV=leucovorin; CR=Complete response; PR=partial response; NC=no change; PD=progressive disease; RR=risk ratio; CI=confidence interval.

**Table 3:** Exploratory analysis of effects of prognostic factors on clinical outcome

**End point**	**Factor**	**RR (95% CI)**
Response	Peritoneal involvement:	0.43
	Yes *vs* no	(0.13–1.47)
	Surgery of primary tumour:	1.78
	Yes *vs* no	(0.76–4.15)
	Baseline Karnofsky status:	0.41^*^
	⩽80 *vs* >80	(0.17–0.95)
	Treatment effect:	0.42^*^
	ELF *vs* ILF	(0.18–0.97)
		
Progression	Peritoneal involvement:	1.15
	Yes *vs* no	(0.67–1.96)
	Surgery of primary tumour:	0.86
	Yes *vs* no	(0.58–1.29)
	ELF *vs* ILF	1.7
	(in patients with Karnofsky status ⩽80)	(0.96–3.0)
	ELF *vs* ILF	0.8
	(in patients with Karnofsky status >80)	(0.47–1.39)
	Number of organs:	1.99^**^
	>1 *vs* 1	(1.21–3.28)
	Site of primary tumour:	1.91^**^
	oesophagogastric *vs* stomach	(1.2–3.04)
		
Death	Peritoneal involvement:	0.85
	Yes *vs* no	(0.42–1.75)
	Surgery of primary tumour:	0.69
	Yes *vs* no	(0.46–1.06)
	ELF *vs* ILF	2.41
	(in patients with peritoneal involvement)	(0.99–5.82)
	ELF *vs* ILF	1.01
	(in patients without peritoneal involvement)	(0.64–1.59)
	Number of organs:	2.56^**^
	>1 *vs* 1	(1.48–4.42)
	Baseline Karnofsky status:	1.84^**^
	⩽80 *vs* >80	(1.21–2.8)

RR=risk ratio; CI=confidence interval.

A risk value >1 shows an increased likelihood of the clinical outcome in favour of the first of the two compared terms. ELF, etoposide+LV (leucovorin)+5-FU(5-fluorouracil); ILF, irinotecan+LV+5-FU; EJ, oesophagogastric junction.

^*^P<0.05.

^**^<0.01 by Wald *χ*^2^ test.

**Table 4:** Survival

	**Irinotecan+5- FU/LV (*N*=56)**	**Etoposide+5- FU/LV (*N*=58)**	
**Survival parameter**	**Median time in months (95% CI)**		**Statistical comparison^a^ Hazard ratio (95% CI)**
Progression-free survival	4.5 (3.4–5.8)	2.3 (2.0–4.7)	1.10 (0.75–1.62)
			*P*=0.6116
Time to treatment failure	3.6 (2.4–5.1)	2.2 (1.5–2.9)	1.15 (0.79–1.67)
			*P*=0.4542
Overall survival	10.8 (9.0–13.2)	8.3 (6.6–11.4)	1.25 (0.83–1.86)
			*P*=0.2818

5-FU=5-fluorouracil; LV=leucovorin; CI=confidence interval.

a Hazard ratio >1 favors irinotecan+5-FU/LV; *P*-value from log-rank test.

**Table 5:** Dosing information

	**Irinotecan+5- FU/LV (*N*=56)**	**Etoposide+5- FU/LV (*N*=58)**
Median number of cycles (range)	2 (0–14)	3 (1–19)
Number of cycles administered as planned	33 (59%)	48 (82%)
Median months of treatment duration (range)	3.6 (0–22)	1.5 (0–15)
Number (%) of administration delays	39 (70)	30 (52)
Number (%) of dose reductions	42 (75)	26 (45)

5-FU=5-fluorouracil; LV=leucovorin.

**Table 6:** Grade 3 or 4 toxicity according to NCIC-CTC

	**Irinotecan+5- FU/LV (*N*=56)**	**Etoposide+5- FU/LV (*N*=58)**
**Adverse event category**	**Percentage of patients in each category**	
*Haematological toxicity*		
Anaemia	7	9
Neutropenia	9	57
Thrombocytopenia	4	5
Infection with neutropenia	4	3
Fever+neutropenia, no infection	2	3
		
*Gastrointestinal toxicity*		
Nausea	16	7
Diarrhoea	18	0
Vomiting	7	5
		
*Other toxicity*		
Infection without neutropenia	2	7
Anorexia	2	7
Alopecia	5	28
Constipation	2	0

NCIC-CTC=National Cancer Institute of Canada expanded common toxicity criteria; 5-FU=5-fluorouracil; LV=leucovorin.
